# Association between Dietary Inflammatory Index and Periodontitis: A Cross-Sectional and Mediation Analysis

**DOI:** 10.3390/nu13041194

**Published:** 2021-04-05

**Authors:** Vanessa Machado, João Botelho, João Viana, Paula Pereira, Luísa Bandeira Lopes, Luís Proença, Ana Sintra Delgado, José João Mendes

**Affiliations:** 1 Clinical Research Unit (CRU), Centro de Investigação Interdisciplinar Egas Moniz (CiiEM), Egas Moniz–Cooperativa de Ensino Superior, 2829-511 Almada, Portugal; vmachado@egasmoniz.edu.pt (V.M.); jpm.viana.1@gmail.com (J.V.); luisabandeiralopes@gmail.com (L.B.L.); anasintradelgado@gmail.com (A.S.D.); jmendes@egasmoniz.edu.pt (J.J.M.); 2 Evidence-Based Hub, Clinical Research Unit, Centro de Investigação Interdisciplinar Egas Moniz, 2829-511 Almada, Portugal; lproenca@egasmoniz.edu.pt; 3 Grupo de Estudos em Nutrição Aplicada (GENA), CiiEM, Egas Moniz–Cooperativa de Ensino Superior, 2829-511 Almada, Portugal; pereira.paula1@gmail.com; 4 Quantitative Methods for Health Research (MQIS), CiiEM, Egas Moniz–Cooperativa de Ensino Superior, 2829-511 Almada, Portugal

**Keywords:** periodontitis, periodontal disease, inflammation, diet, oral health

## Abstract

Inflammation-modulating elements are recognized periodontitis (PD) risk factors, nevertheless, the association between dietary inflammatory index (DII) and PD has never been appraised. We aimed to assess the association between DII and PD and the mediation effect of DII in the association of PD with systemic inflammation. Using the National Health and Nutrition Examination Survey 2009–2010, 2011–2012 and 2013–2014, participants who received periodontal exam and provided dietary recall data were included. The inflammatory potential of diet was calculated via DII. PD was defined according to the 2012 case definition. White blood cells (WBC), segmented neutrophils and C-reactive protein (CRP) were used as proxies for systemic inflammation. The periodontal measures were regressed across DII values using adjusted multivariate linear regression and adjusted mediation analysis. Overall, 10,178 participants were included. DII was significantly correlated with mean periodontal probing depth (PPD), mean clinical attachment loss (CAL), thresholds of PPD and CAL, WBC, segmented neutrophils and DII (*p* < 0.01). A linear regression logistic adjusted for multiple confounding variables confirmed the association between DII and mean PPD (B = 0.02, Standard Error [SE]: 0.02, *p* < 0.001) and CAL (B = −0.02, SE: 0.01, *p* < 0.001). The association of mean PPD and mean CAL with both WBC and segmented neutrophils were mediated by DII (from 2.1 to 3.5%, *p* < 0.001). In the 2009–2010 subset, the association of mean CAL with serum CRP was mediated by DII (52.0%, *p* < 0.01). Inflammatory diet and PD may be associated. Also, the inflammatory diet significantly mediated the association of leukocyte counts and systemic inflammation with PD.

## 1. Introduction

Life expectancy is heavily dependent on diet [1,2,3,4]. The inflammatory burden of diet has brought up great interest considering the anti- and proinflammatory modulating potential of nutrients. Proinflammatory dietary patterns have been associated with some systemic conditions, including cardiovascular diseases [5,6], higher mortality rate in breast cancer survivors [7], rheumatoid arthritis [8] or periodontal disease [9]. Further, the systemic balance of chronic inflammation is more dependent on diet than medication usage, given the frequency of food ingestion [10]. Thus, if the status quo of inflammation is controlled, several reactions or diseases determined by inflammatory pathways may be avoided or interrupted. To a certain extent, the inflammatory load of diet fosters a more comprehensive outlook rather than single-nutrient based assessments and with considerable research interest [11]. For this reason, the Dietary Inflammatory Index (DII) was proposed as a literature-based tool to categorize individuals’ diets based on global standard values [12,13]. Ever since, DII has been associated with the variation of inflammatory markers [14,15,16] and implicated in systemic diseases [17,18] and tooth loss [19], but never in periodontitis (PD).

PD is a pandemic condition characterized by a progressive loss of integrity of tissues supporting teeth, gingival bleeding and halitosis [20,21], and ultimately tooth loss [22]. This non-communicable disease results from an accumulation of oral biofilm around the teeth, causing an exacerbated reaction from the immune system that destroys the periodontium [23]. PD starts with gingivitis (gum inflammation without bone loss) however, if untreated, can precipitate bone loss and, ultimately, tooth loss [24]. Besides, PD is responsible for a reversible impairment of patient’s quality of life [25,26]. Some studies demonstrate a link between periodontal disease and nutrition [27,28,29]. In particular, anti-inflammatory diet demonstrated therapeutical potential in gingivitis [9], still, regular flavonoid intake, recognized anti-inflammatory compounds, had no association with the risk of PD [30]. To date, no study has explored the relation between DII and the periodontal status, and such association might have significant value to oral and systemic health.

The present study explored whether the inflammatory degree of diet, measured via DII, is associated with PD. Secondly, we investigated the mediation effect of DII in the association of PD with systemic inflammation (assessed through white blood cells count [WBC], segmented neutrophils and C-reactive protein [CRP]). We hypothesized that worse periodontal clinical measures are associated with the inflammatory load originated in the diet, and that this load might significantly mediate the systemic inflammatory burden caused by PD.

## 2. Materials and Methods

### 2.1. Study Design and Participants

This secondary analysis used data from the National Health and Nutrition Examination Survey (NHANES) collected between 2009–2010, 2011–2012 and 2013–2014. The NHANES is a stratified multistage research program conducted by the National Center for Health Statistics (NCHS) of the civilian non-institutionalized population in the 50 stages of the United States (US) and the District of Columbia. NHANES aims to assess the health and nutritional status and to monitor changes over time. The way of collecting data is through interviews, physical and laboratory exams, and all NHANES 2009–2014 were reviewed and approved by the Centers for Disease Control (CDC) and Prevention National Center for Health Statistics Research (NCHS) Ethics Review Board, and all included participants provided written informed consent.

For the purpose of this study, NHANES 2009–2010; 2011–2012 and 2013–2014 data were retrieved with the following inclusion criteria: participants aged 18 or over, who received periodontal examination and answered the dietary interview for the total nutrient intakes. We followed the extension of the STrengthening the Reporting of OBservational studies in Epidemiology for Nutritional Epidemiology (STROBE-Nut) [31] (Table S1).

### 2.2. Diet. and Dietary Inflammatory Index

The dietary interview data carried out on the NHANES participants were used to estimate the types and amounts of foods and beverages consumed during the 24 h period prior the interview (midnight to midnight).

The DII is a literature-based tool designed to categorize individuals’ diets on a continuum from maximally anti-inflammatory to maximally pro-inflammatory [12,13]. Briefly, the DII is a scoring algorithm based on dietary intake data that are linked to a regionally representative world database to estimate a mean and standard deviation (SD) for each parameter. In this study, from a possible total of 46 food parameters, we calculated the DII score for 26 food parameters available: energy (kcal), protein (gm), carbohydrate (gm), total sugar (gm), dietary fiber (gm), total fat (gm), total saturated fatty acids (gm), total monosaturated fatty acids (gm), total polyunsaturated fatty acids (gm), cholesterol (mg), vitamin E as α-tocopherol (mg), eugenol, garlic, ginger, niacin, onion, saffron, saturated fat, trans fat, turmeric, green/black tea, flavan-3-ol, flavones, flavonols, flavonones, anthocyanidins, isoflavones, pepper, thyme/oregano, rosemary were not included because no information was available. First, we calculated the z-score of each food parameter and for each participant. Second, each individual z-score was converted to a centered percentile. Third, each centered percentile was multiplied by the standardized overall inflammatory effect score [12]. Then, the DII score was summed for each participant. The final score is a continuous measure, interpreted as strongly anti-inflammatory (the lowest score) to strongly proinflammatory (the highest score), respectively [12].

### 2.3. Periodontal Assessment

Full-month periodontal examination at six sites per tooth (mesiobuccal, buccal, distobuccal, mesiolingual, lingual, and distolingual) was conducted by trained calibrated examiners as described elsewhere [32]. Probing pocket depth (PPD) and gingival recession measures were made using a HU-Friedy periodontal probe graduated in 2 mm increments. Clinical attachment loss (CAL) was calculated as the difference between PPD and gingival recession. The PD diagnosis and staging were carried out following the CDC and Prevention-American Academy of Periodontology consensus for epidemiologic studies recommendation [33].

### 2.4. Covariates 

Self-reported socio-demographic characteristics regarding age, gender, race or ethnicity (i.e., Mexican American, Non-Hispanics White, Non-Hispanics Black, other Hispanics and other races) and higher education level (i.e., less than high school, complete high school or similar, higher than high school) was collected for each wave. Smoking status was subdivided as never (smoked less than 100 cigarettes in life and not currently smoking), former (smoked at least 100 cigarettes in life and not currently smoking), active smoker (smoked at least 100 cigarettes in life and currently smoking). Body mass index (BMI) was calculated as weight in kilograms divided by height in meters squared. Systolic and diastolic blood pressure (SBP and DBP) measurements were determined by trained and calibrated examiners. Blood levels data included WBC Count (10^9^/L), segmented neutrophils (10^9^/L), vitamin D (mg/dL), hemoglobin A1c (Hba1c) (%), total cholesterol and CRP (mg/dL).

### 2.5. Statistical Analysis

We collected data from the NHANES 2009–2010; 2011–2012 and 2013–2014 were uploaded through SAS Universal Viewer for Windows and handled with IBM SPSS Statistics version 25.0 for Macintosh (IBM Corp., Armonk, NY, USA) To make the periodontal diagnosis, we export our data to a Microsoft Excel spreadsheet. Descriptive measures are reported through mean (SD) for continuous variables, and a number of cases (n) and percentage (%) for categorical variables.

The correlation between the DII and CRP with the periodontal clinical measures, WBC and segmented neutrophils was assessed by the Spearman correlation coefficient. Further, a multivariate stepwise adjusted linear regression procedure was used to model the influence of DII and CRP on the periodontal clinical measures, WBC and segmented neutrophils. Model variables were selected among clinical and demographic characteristics. Following the initial crude model (model 1), seven progressively adjusted models were generated (model 2—includes adjustment for DII and age; model 3—includes adjustment for DII, age and BMI; model 4—includes adjustment for DII, age, BMI and number of chronic medical conditions; model 5—includes adjustment for DII, age, BMI, number of chronic medical conditions and SBP; model 6—includes adjustment for DII, age, BMI, number of chronic medical conditions, SBP and Hba1c; model 7—includes adjustment for DII, age, BMI, number of chronic medical conditions, SBP, Hba1c and total cholesterol; model 8—includes adjustment for DII, age, BMI, number of chronic medical conditions, SBP, Hba1c, total cholesterol and vitamin D levels).

**Figure 1:**
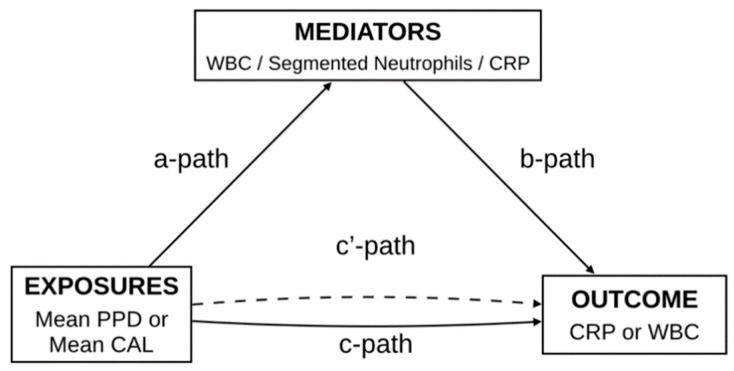
Path diagram of the mediation analysis models. CAL—Clinical Attachment Loss; CRP—C-reactive protein; PPD—Periodontal probing depth; WBC—White blood cells counts.

The mediation analyses were carried out using the r package ‘lavaan’. To investigate the potential mediating effect of DII on the association between mean PPD or mean CAL (exposure) and CRP/WBC (outcome) we used multivariable regression analysis. Three pathways (a, b and c) were used to assess the mediation (Figure 1). Total effect evaluated the relationship between mean CAL/PPD (exposure) and CRP/WBC (outcome). Path a assessed the relationship between mean CAL/PPD and DII (mediator). Path b measured the relationship between DII (mediator) and CRP/WBC (outcome). The influence of DII on the link between mean CAL/PPD and CRP/WBC were assessed through path c (direct effect). The proportion of the mediated effect was calculated using the following formula: (total effect-direct effect) × 100/βtotal effect. All models were conducted adjusted for sociodemographic variables (age, gender, race, education level), health behaviors (minutes of sedentarism, smoking habit, alcohol use), BMI, number of missing teeth and systemic status (number of medical conditions, SBP, DBP, Hba1c, total cholesterol and vitamin D). A value of *p* < 0.05 was considered significant.

## 3. Results

### 3.1. Characteristics of the Study Sample

From an initial sample of 30,468 participants, 20,290 were excluded considering the they had not received periodontal examination, resulting in a final sample of 10,178 patients.

The characteristics of this sample are depicted in Table 1. The overall sample had an average age of 52.0 (14.3) years and mostly were female participants (50.7%), and there was a higher incidence of PD levels in middle-aged individuals. This sample was predominantly composed by Non-Hispanic White (30.7%) and Non-Hispanic Black (30.4%) participants with higher education level (55.2%). Regarding smoking habits, most of the participants never smoked (56.2%), and 18.6% were active smokers. Also, this sample had an average BMI of 29.1 (7.0) Kg/m^2^ and high prevalence of PD (59.4%). Particularly, CRP values were only available for the sample of 2009–2010 NHANES.

### 3.2. DII, PD and Systemic Inflammation

We investigated the correlation of DII and CRP with periodontal measures, WBC and segmented neutrophils (Table 2). In the overall sample, DII was significantly correlated with mean PPD, mean CAL, thresholds of PPD and CAL, WBC and segmented neutrophils (*p* < 0.01). Additionally, CRP had a significant correlation with mean PPD, mean CAL, thresholds of PPD and CAL, WBC, segmented neutrophils and DII (*p* < 0.01).

Next, we examined the linear association of DII in a crude (model 1) and adjusted models (models 2–8) for mean PPD, mean CAL, n sites PPD ≥ 5 and CAL ≥ 5 (Table 3). Linear regression models confirmed that DII is significantly associated with mean PPD (ß coefficient = −0.06, SE = 0.01, *p* < 0.001), mean CAL (ß coefficient = −0.08, SE = 0.01, *p* < 0.001), n sites PPD ≥ 5 (ß coefficient = −0.01, SE = 0.00, *p* < 0.001) and n sites CAL ≥ 5 (ß coefficient = −0.02, SE = 0.00, *p* < 0.001).

After confirming the aforementioned multivariate associations, we constructed a mediation model to explore the relationship between one independent variable (WBC, segmented neutrophils, CRP) and two dependent variables (mean PPD and mean CAL) for the overall sample (Table 4), and one independent variable for the NHANES 2009–2010 (CRP) (Table 5). As a mediator, we considered the DII score. All mediation analyses were computed adjusted for sociodemographic variables (age, gender, race, education), health behaviors (minutes of sedentarism, smoking habit, alcohol use), BMI, and systemic status (number of medical conditions, SBP, DBP, Hba1c, total cholesterol, vitamin D). A statistically significant model was observed when DII was included as a mediator of the association between mean PPD and segmented neutrophils. An indirect effect (3.5%) of mean PPD on segmented neutrophils mediated by DII was confirmed (β = 0.00, SE = 0.00) (Table 4). Similarly, a statistically model was obtained when DII was considered as a mediator in the association of mean CAL with WBC, with a statistically significant indirect effect 2.1%. Further, mean CAL mediated by DII had a significant indirect effect in CRP level (52.0%) (Table 5).

## 4. Discussion

Overall, in this cross-sectional study of American participants in a national representative survey, the dietary inflammatory load and periodontitis might be linked. Also, the dietary inflammatory level consistently mediated the association between periodontal measures and levels of systemic inflammation.

Until now, only one study had employed the DII tool to appraise the association of inflammatory diet with oral problems, as an anti-inflammatory diet was associated with less missing teeth [19]. Interestingly, the sample included in Kotsakis et al. [19] (NHANES 2009–2010 and 2011–2012) was also included in our study, though we also added the NHANES wave of 2013–2014. For this reason, the number of missing teeth was accounted as a confounding variable to the adjusted analyses. Hence, this study is the first to employ the DII instrument in such a comprehensive and large group of patients diagnosed with PD.

The scientific quest on how nutrition impacts periodontal health has gained wide interest. Dietary behavior was proposed to affect periodontal inflammation in a clinically relevant fashion [34]. Despite the fact that anti- or pro-inflammatory diets are still poorly studied in periodontal research, several other diet types that encompass inflammatory components have been studied in the past. For instance, individuals that consume a Mediterranean diet, described as anti-inflammatory, were associated with lower levels of periodontal bacteria [35], yet this dietary pattern had no effect on insulin resistance in PD patients [36]. Furthermore, probiotic-rich diet presented contradictory effects on periodontal disease [37,38,39] and vegan diet did not demonstrate a direct relationship with PD despite influencing salivary microbiota [40]. Regarding particular foods, high-intake doses of cocoa were associated with reduced levels of oxidative stress in PD cases [41]. However, lack of consistency regarding the inflammatory load of diet may explain previous inconsistent results and support the importance of using a comprehensive tool as DII in epidemiological and clinical settings.

A landmark investigation demonstrated that gum bleeding consistently decreased (from 34.8% to 12.6% of the overall mouth) after 4 weeks under a regimen of no oral hygiene and no processed diet intake. The lack of oral hygiene led to significant plaque accumulation, yet the absence of processed diet consumption may have contributed to low gingival inflammation [42], however, this study lacked a control group to confirm the observed association. Later, a 4-week low-carbohydrate diet (rich in omega-3 fatty acids, vitamin C and D, antioxidants and fiber) reduced gingival and periodontal inflammation, in a randomized controlled trial [43]. In a recent controlled trial, a 4-week anti-inflammatory diet significantly improved gingivitis but had no impact on the microbiome composition and serum levels of inflammation [9]. Nonetheless, the results provided by these trials emphasize the potential of combining appropriate oral health routines with low-inflammatory diets.

From a systemic perspective, a pro-inflammatory diet contributes to higher values of systemic inflammation [44]. In fact, DII is a tool that consistently reflects the levels of six inflammatory markers: interleukin (IL)-1β, IL-4, IL-6, IL-10, TNF-α and CRP [12]. The possible biological mechanism that links diet and CRP is hypothesized to be based on the components of the inflammatory diet that indirectly promote the activation of CRP, thus leading to greater production of this protein [15,16,45]. In detail, the consumption of the so-called pro-inflammatory elements causes an increase in circulating IL levels (particularly IL-6, IL-1β or IL-8) [46]. In response, CRP is dramatically produced in hepatic cells and released into circulation, fostering an even more inflamed systemic status [46].

On the other hand, PD causes a local inflammatory reaction that can evolve systemically, increasing WBC and segmented neutrophil counts [47,48]. These types of leukocytes are responsible to be in the frontline to fight periodontal infection [49]. If this local infection persists, the bone marrow is signalized to produce a higher number of inflammatory cells [50]. If the periodontal epithelium turns ulcerated, periodontal pathogens may invade the organism and trigger a systemic response to neutralize any harmful consequences [23]. Additionally, clinical situations in which proinflammatory levels are increased (CRP, TNF-α or interleukins) incite the host immune to react against periodontal infection exacerbating the periodontal destruction [51]. Therefore, mediators of inflammation play a key role in the progression of PD, and our results support it regarding DII via the average values of CAL.

In this way, we might hypothesize that PD patients consuming a highly inflammatory diet can not only aggravate clinical symptoms but also be associated with an increased risk towards other relevant systemic conditions, such cardiovascular disease and diabetes where PD plays a key role as a modifying factor [52,53]. Both PD and DII present direct links to systemic conditions, but indirectly they might convey to worse systemic status.

We also judged from the analysis of our results that patients who have PD when subjected to an anti-inflammatory diet might demonstrate positive results in involution in the disease, though this warrants confirmation through randomized clinical trials in PD patients.

### Strengths and Limitations

This study has some limitations. The cross-sectional survey nature does not allow any inference of causality or temporal relationship between the PD and DII. A further limitation is the fact that our analyses were based on dietary self-reported questionnaires, and thereby there is an inherent accepted bias. Also, the DII was computed from 26 food parameters (out of possible 45) from the NHANES questionnaire, yet a previous study has performed this alike [19,54]. Also, the questionnaire refers to a 24-h period prior to the interview and this may be seen as a shortcoming [16].

Furthermore, the relatively young age of the studied population is a matter to consider. Age is a known risk factor for the severity of PD, and therefore, future studies should account for the relationship between DII and elder populations suffering from PD. Additionally, the available periodontal data lacked measures of local inflammation, such as bleeding on probing or periodontal inflamed surface area.

Another important point is the shortage information on systemic inflammation markers, as the CRP levels were only available on NHANES 2009–2010, which limits the validity of these results. Also, data on TNF-α and interleukins would be of interest to study in future research. When exploring the association and mediated effect, these results should be interpreted cautiously, as the causal association will only be possible to prove through prospective studies and randomized intervention trials.

Nevertheless, a number of strengths are worth mentioning. This large sample is based on three consecutive NHANES waves, representative of the American population. The number of covariates included in our analyses were comprehensive and provided consistency to the results. The periodontal assessment was made by calibrated examiners and on a full-mouth basis, increasing the accuracy and precision of the results [55,56].

## 5. Conclusions

Inflammatory diet and PD may be associated, as patients with a more pro-inflammatory dietary pattern are more likely to present worse periodontal measures. Also, the inflammatory diet significantly mediated the association of leukocyte counts and systemic inflammation with PD.

## Figures and Tables

**Table 1:** Sample characteristics (*n* = 10,178).

	NHANES 2009–2010 (*n* = 3238)	NHANES 2011–2012 (*n* = 3323)	NHANES 2013–2014 (*n* = 3617)	NHANES 2009–2014 (*n* = 10,178)
Age (years), mean (SD)	52.2 (14.4)	51.8 (14.2)	52.0 (14.3)	52.0 (14.3)
Gender, *n* (%)				
Males	1623 (50.1)	1643 (49.4)	1752 (48.4)	5018 (49.3)
Females	1615 (49.9)	1680 (50.6)	1865 (51.6)	5160 (50.7)
Race/ethnicity, *n* (%)				
Mexican American	585 (18.1)	355 (10.7)	491 (13.6)	1431 (14.1)
Non-Hispanic White	339 (10.5)	1226 (36.9)	1562 (43.2)	3127 (30.7)
Non-Hispanic Black	1555 (48.0)	839 (25.2)	704 (19.5)	3098 (30.4)
Other Hispanic	586 (18.1)	343 (10.3)	322 (8.9)	1251 (12.3)
Other race	173 (5.3)	560 (16.9)	538 (14.9)	1271 (12.5)
Education level, *n* (%)				
<High school	401 (12.4)	318 (9.6)	283 (7.9)	1000 (9.8)
High school	1203 (37.2)	1135 (34.2)	1217 (33.7)	3555 (34.9)
>High school	1634 (50.5)	1870 (56.2)	2117 (58.5)	5623 (55.2)
Smoking status, *n* (%)				
Never	1777 (54.9)	1900 (57.2)	2046 (56.6)	5723 (56.2)
Former	841 (26.0)	813 (24.5)	905 (25.0)	2559 (25.1)
Current	620 (19.1)	610 (18.4)	666 (18.4)	1896 (18.6)
BMI (kg/m^2^), mean (SD)	29.4 (6.5)	28.8 (27.9)	29.2 (7.3)	29.1 (7.0)
Blood pressure, mean (SD)				
SBP (mmHg)	115.7 (29.1)	124.2 (18.0)	124 (17.7)	121.8 (22.5)
DBP (mmHg)	66.5 (18.5)	72.3 (11.3)	71.4 (11.1)	70.1 (14.2)
Periodontitis, n (%)	2065 (63.8)	2076 (62.5)	1900 (52.5)	6041 (59.4)
Missing teeth, mean (SD)	6.3 (6.1)	5.4 (6.3)	5.5 (6.2)	5.7 (6.2)
DII, mean (SD)	−0.27 (1.80)	−0.21 (1.82)	−0.53 (1.98)	−0.35 (1.88)
Blood levels, mean (SD)				
WBC (109/L)	6.7 (3.1)	6.6 (2.4)	7.1 (2.5)	6.9 (2.6)
Segmented neutrophils (109/L)	4.0 (2.6)	3.94 (1.8)	4.2 (1.8)	4.1 (1.9)
Hba1c (%)	5.6 (1.6)	5.6 (1.6)	5.7 (1.3)	5.7 (1.4)
Vitamin D (mg/dL)	64.5 (25.6)	62.8 (30.9)	67.1 (28.7)	64.7 (28.7)
Total Cholesterol	200.2 (40.0)	186.9 (60.6)	190.0 (49.9)	191.5 (53.1)
CRP (mg/dL)	0.39 (0.74)	-	-	-

BMI—Body Mass Index; SBP—Systolic Blood Pressure; DBP—Diastolic Blood Pressure; PPD—Periodontal probing depth; CAL—Clinical Attachment Loss; WBC—White blood cells counts; DII—Dietary Inflammatory Index; Hba1c—Glycated Hemoglobin a1c; CRP—C-reactive Protein.

**Table 2:** Correlation between DII score with periodontal clinical measures, circulating leukocyte levels for the overall sample (*n* = 10,178) and C-Reactive Protein for the NHANES 2009–2010 sample (*n* = 3238).

	NHANES 2009–2014 (*n* = 10,178)	NHANES 2009–2010 (*n* = 3238)
Variable	DII	CRP
Mean PPD (mm)	−0.047 **	0.055 **
Mean CAL (mm)	−0.042 **	0.062 **
n sites PPD ≥ 5 mm (%)	−0.039 **	0.070 **
n sites PPD ≥ 7 mm (%)	−0.030 **	0.056 **
n sites CAL ≥ 5 mm (%)	−0.037 **	0.046 **
n sites CAL ≥ 7 mm (%)	−0.030 **	0.068 **
WBC (10^9^/L)	−0.046 **	0.221 **
Segmented neutrophils (10^9^/L)	−0.039 **	0.232 **
DII	-	0.103 **

Spearman correlation, ** *p* < 0.01. PPD—Periodontal probing depth; CAL—Clinical Attachment Loss; WBC—White blood cells counts; DI—Dietary Inflammatory Index; CRP—C-reactive Protein.

**Table 3:** Crude and adjusted linear regression models and correspondent standard error towards mean PPD, mean CAL, PPD ≥ 5 and CAL ≥ 5 with DII for the overall samples (*n* = 10,178).

NHANES 2009—2014 (*n* = 10,178)	Mean PPD (mm)	Mean CAL (mm)	n sites PPD ≥ 5 mm (%)	n sites CAL ≥ 5 mm (%)
Model 1	0.02 (0.02) ***	−0.02 (0.01) ***	0.00 (0.00) ***	0.00 (0.00) ***
Model 2	0.02 (0.00) ***	−0.02 (0.01) ***	0.00 (0.00) ***	0.00 (0.00) ***
Model 3	0.02 (0.00) ***	−0.02 (0.01) ***	0.00 (0.00) ***	0.00 (0.00) ***
Model 4	0.02 (0.00) ***	−0.02 (0.01) ***	0.00 (0.00) ***	0.00 (0.00) ***
Model 5	0.02 (0.00) ***	−0.02 (0.01) ***	0.00 (0.00) ***	0.00 (0.00) ***
Model 6	0.02 (0.00) ***	−0.02 (0.01) ***	0.00 (0.00) ***	0.00 (0.00) ***
Model 7	0.02 (0.00) ***	−0.02 (0.01) ***	0.00 (0.00) ***	0.00 (0.00) ***
Model 8	0.02 (0.00) ***	−0.02 (0.01) ***	0.00 (0.00) **	0.00 (0.00) ***

Model 1—Unadjusted model for dietary inflammatory index (DII); Model 2—Includes adjustment for DII and age; Model 3—Includes adjustment for DII, age and body mass index (BMI); Model 4—Includes adjustment for DII, age, BMI and number of chronic medical conditions; Model 5—Includes adjustment for DII, age, BMI, number of chronic medical conditions and systolic blood pressure (SBP); Model Includes adjustment for DII, age, BMI, number of chronic medical conditions, SBP and hemoglobin A1c (Hba1c); Model 7—Includes adjustment for DII, age, BMI, number of chronic medical conditions, SBP, Hba1c and total cholesterol; Model 8—Includes adjustment for DII, age, BMI, number of chronic medical conditions, SBP, Hba1c, total cholesterol and vitamin D levels. ** *p* < 0.01; *** *p* < 0.001. PPD—Periodontal probing depth; CAL—Clinical Attachment Loss.

**Table 4:** Mediation analysis of the effects of systemic inflammatory elements on the association of periodontal measures (mean PPD and mean CAL—Exposures) with white blood cells and segmented neutrophils (*n* = 10,178).

	Path a	Path b	Path c (Direct Effect)	Mediated Effect	Total Effect	Proportion Mediated (%)
**Exposure**: Mean PPD						
WBC (10^9^/L)	0.14 (0.04) ***	0.04 (0.02) ***	0.21 (0.06) ***	0.01 (0.00)	0.21 (0.06) ***	2.7
Segmented Neutrophils (10^9^/L)	0.14 (0.04) ***	0.04 (0.02) ***	0.15 (0.04) ***	0.01 (0.00)	0.16 (0.04) ***	3.5
**Exposure**: Mean CAL						
WBC (10^9^/L)	0.10 (0.03) ***	0.04 (0.02) ***	0.17 (0.04) ***	0.01 (0.00)	0.17 (0.04) ***	2.4
Segmented Neutrophils (10^9^/L)	0.10 (0.03) ***	0.03 (0.02) *	0.13 (0.03) ***	0.01 (0.00)	0.14 (0.03) ***	2.1

All models were conducted adjusted for sociodemographic variables (age, gender, race, education), health behaviors (minutes of sedentarism, smoking habit, alcohol use), body mass index, number of missing teeth and systemic status (number of medical conditions, systolic blood pressure, diastolic blood pressure, hemoglobin A1c, total cholesterol, vitamin D). * *p* < 0.05; ** *p* < 0.01; *** *p* < 0.001. Abbreviations: CAL—clinical attachment loss; DII—dietary inflammatory index, PPD—Periodontal Pocket Depth; WBC—white blood cells. 95% CI—95% confidence interval. 5000 number of bootstrap samples. WBC—White Blood Cells; PPD—Periodontal probing depth; CAL—Clinical Attachment Loss.

**Table 5:** Mediation analysis of the effects of systemic inflammatory elements on the association of periodontal measures (mean PPD and mean CAL–Exposures) with C-reactive protein (*n* = 3238).

	Path a	Path b	Path c (Direct Effect)	Mediated Effect	Total Effect	Proportion Mediated (%)
**Exposure**: Mean PPD						
CRP (mg/dL)	0.09 (0.07)	0.04 (0.01) **	0.08 (0.04)	0.01 (0.00)	0.07 (0.04) *	NA
**Exposure**: Mean CAL						
CRP (mg/dL)	0.13 (0.05) *	0.04 (0.01) **	0.06 (0.03) *	0.01 (0.00)	0.07 (0.03) **	52.0

All models were conducted adjusted for sociodemographic variables (age, gender, race, education), health behaviors (minutes of sedentarism, smoking habit, alcohol use), body mass index, number of missing teeth and systemic status (number of medical conditions, systolic blood pressure, diastolic blood pressure, hemoglobin A1c, total cholesterol, vitamin D). Abbreviations: CAL—Clinical Attachment Loss; DII—Dietary Inflammatory Index, PPD—Periodontal Pocket Depth. 95% CI—95% Confidence Interval. * *p* < 0.05; ** *p* < 0.01; *** *p* < 0.001. 5000 number of bootstrap samples. CRP—C-reactive Protein; PPD—Periodontal probing depth; CAL—Clinical Attachment Loss.

## Data Availability

Data is available at NHANES website.

## Supplementary Materials

The following are available online at https://www.mdpi.com/article/10.3390/nu13041194/s1, Table S1: STROBE-Nut checklist.
